# The role of biomechanical forces and MALAT1/miR‐329‐5p/PRIP signalling on glucocorticoid‐induced osteonecrosis of the femoral head

**DOI:** 10.1111/jcmm.16510

**Published:** 2021-05-03

**Authors:** Guomin Li, Bing Li, Bo Li, Jie Zhao, Xiaoquan Wang, Rui Luo, Yankun Li, Jun Liu, Ruyin Hu

**Affiliations:** ^1^ Department of Orthopedics Guizhou Provincial People’s Hospital Guiyang China; ^2^ Department of Joint Tianjin Hospital Tianjin China

**Keywords:** biomechanical forces, glucocorticoid‐induced osteonecrosis of the femoral head, MALAT1, miR‐329‐5p, PRIP

## Abstract

Glucocorticoid‐induced osteonecrosis of the femoral head (GIONFH) is a common orthopaedic disease. GIONFH primarily manifests clinically as hip pain in the early stages, followed by the collapse of the femoral head, narrowing of the hip joint space and damage to the acetabulum, resulting in severely impaired mobility. However, the pathogenesis of GIONFH is not clearly understood. Recently, biomechanical forces and non‐coding RNAs have been suggested to play important roles in the pathogenesis of GIONFH. This study aimed to evaluate the role of biomechanical forced and non‐coding RNAs in GIONFH. We utilized an in vivo, rat model of GIONFH and used MRI, μCT, GIONFH‐TST (tail suspension test), GIONFH‐treadmill, haematoxylin and eosin staining, qRT‐PCR and Western blot analysis to analyse the roles of biomechanical forces and non‐coding RNAs in GIONFH. We used RAW264.7 cells and MC3T3E1 cells to verify the role of MALAT1/miR‐329‐5p/PRIP signalling using a dual luciferase reporter assay, qRT‐PCR and Western blot analysis. The results demonstrated that MALAT1 and PRIP were up‐regulated in the femoral head tissues of GIONFH rats, RAW264.7 cells, and MC3T3E1 cells exposed to dexamethasone (Dex). Knockdown of MALAT1 decreased PRIP expression in rats and cultured cells and rescued glucocorticoid‐induced osteonecrosis of femoral head in rats. The dual luciferase reporter gene assay revealed a targeting relationship for MALAT1/miR‐329‐5p and miR‐329‐5p/PRIP in MC3T3E1 and RAW264.7 cells. In conclusion, MALAT1 played a vital role in the pathogenesis of GIONFH by binding to (‘sponging’) miR‐329‐5p to up‐regulate PRIP. Also, biomechanical forces aggravated the pathogenesis of GIONFH through MALAT1/miR‐329‐5p/PRIP signalling.

## 1 INTRODUCTION

Glucocorticoid‐induced osteonecrosis of the femoral head (GIONFH) is a common orthopaedic disease. The primary early clinical manifestation of GIONFH is hip pain, which is followed by the collapse of the femoral head, narrowing of the hip joint space and damage to the acetabulum, resulting in severely impaired mobility.^1^, ^2^ Glucocorticoids, which are anti‐inflammation and immunosuppressive drugs, are commonly and chronically used in organ transplantation and autoimmune diseases, including systemic lupus erythematosus (SLE), asthma and rheumatoid arthritis (RA).^3^ Based on current reports, the incidence rate for GIONFH ranges from 3% to 38% among individuals taking glucocorticoids in China, whereas in USA, the incidence rate is approximately 40%.^4^, ^5^, ^6^ At present, the pathogenesis of GIONFH is not clearly understood.

Patients with GIONFH in ARCO (Association Research Circulation Osseuse, ARCO)^7^ I and II stages typically are treated clinically by limiting weight‐bearing on the affected hip joint through reduced walking or walking with the aid of crutches. Mont et al^8^ found that limiting weight‐bearing on the affected hip joint delayed the progression of GIONFH and reduced the incidence rate for the collapse of the femoral head (ARCO I 35%, ARCO II 31%, and ARCO III 13%). In 2010, Chinese researchers designed a study that used an SD rat model of GIONFH and provided the rats with treadmill exercise to simulate weight‐bearing movement. The researchers reported that an overweight‐loading condition increased the breakdown of the bone trabeculae, decreased the biomechanical characteristics of the bone and accelerated the pathogenic progression of GIONFH. They also observed glucocorticoid‐induced apoptosis of osteoblasts and osteocytes and the promotion of the differentiation and maturation of osteoclasts. Finally, they concluded that biomechanical forces caused a loss of bone mass, narrowed and increased the brittleness of the bone trabeculae and decreased the biological properties of the femoral head. Thus, these bone changes could easily result in microfractures when biomechanical stress occurred.^9^

Recently, numerous studies have demonstrated that non‐coding RNAs and abnormal bone metabolism participate in the pathogenesis of GIONFH.^10^, ^11^, ^12^ LncRNA MALAT1 and miR‐329‐5p have been documented to have important effects on bone metabolism.^13^, ^14^ Phospholipase C‐related but catalytically inactive protein (PRIP, PLDL or PLCL) was first identified in the cytosol fraction of brain tissue as a novel D‐myo‐inositol 1,4,5‐trisphosphate‐binding protein. PRIP exhibits a domain organization similar to phospholipase C‐δ but lacks phospholipase activity. This similarity is implicated in BMP‐induced osteoblast differentiation by the negative regulation of Smad phosphorylation through the methylation of inhibitory Smad6. Additional evidence is provided by osteoclast differentiation stimulated through calcium‐calcineurin‐NFATc1 signalling by regulating intracellular Ca^2+^ in PRIP‐KO osteoclasts.^15^, ^16^, ^17^

Based on recent advances in elucidating the pathogenic mechanisms of GIONFH, we investigated the effects of biomechanical forces, bone metabolism and non‐coding RNA in glucocorticoid‐induced osteonecrosis of the femoral head. In this study, we observed the microstructure of femoral head in a rat model of GIONFH, before and after exercise on a treadmill or the tail suspension test that results in skeletal unloading. We defined MALAT1/miR‐329‐5p/PRIP signalling using previously published reports and bioinformatics database and assessed signalling changes in the femoral head in the GIONFH rat model using quantitative real‐time PCR (qRT‐PCR) and Western blotting. Finally, we used osteoclast precursor cell‐RAW264.7 and osteoblast precursor cell‐MC3T3E1 cell lines to perform siRNA experiments to verify the signalling pathway.

## 2 MATERIALS AND METHODS

### 2.1 Rat GIONFH model

All animal procedures were conducted according to the National Institutes of Health Guide for the Care and Use of Laboratory Animals, 8th Edition. The study protocol was approved by the Ethics Committee of the Tianjin Hospital and Guizhou Provincial People's Hospital. Male 12‐week‐old Sprague Dawley (SD) rats were housed individually in a temperature‐controlled animal facility with a 12:12 hour light: dark cycle and free access to a commercial rat diet and water.

The glucocorticoid‐induced osteonecrosis of the femoral head (GIONFH) rat model was established based on a protocol reported previously.^18^ Methylprednisolone sodium (500 mg) (MPS: Pfizer) was injected subcutaneously at a dose of 20 mg/kg per day for eight weeks. The unloading model was induced by tail suspension test (TST), in which skeletal unloading was realized through tail suspension, a method described in previous studies.^19^ GIONFH rats were allowed to run on a treadmill (Zhenghua, Anhui, China) at a speed of 1.5 km/h for 0.5 hours, five times a week, to develop the hyper‐weight model. The femoral heads of all the rats were imaged bilaterally using nuclear magnetic resonance scans (GE) beginning in the fourth week.

Euthanasia was performed at the end of each group trial. The right femoral head from each rat was used for μCT imaging and histological evaluation (n = 5/group). The left femoral head from each rat was divided into two parts. One part was used for Western blots and the other part was used for qRT‐PCR (n = 5/group).

### 2.2 Trabecular architecture assessment using μCT

The femoral heads were scanned using an Inveon micro‐PET/CT (Siemens) at a voltage of 80 kV, a current of 500 μA and an entire scan length of 20 mm from the top of the femoral head to the femoral shaft at a spatial resolution of 10 μm. The 3D structures were reconstructed using the Inveon analysis workstation. The range of interest (ROI) was determined as an irregular anatomic contour adjacent to the endocortical surface and epiphyseal line in the proximal epiphysis. The cortical and spongy bone were separated manually using auto‐trace. The trabecular bone and bone marrow were separated using the threshold function (the bone marrow: ≥1800; the trabecular bone: <1800). The bone volume/total volume (BV/TV), bone surface area/bone volume (BS/BV), as well as trabecular thickness, number and separation were calculated.

### 2.3 Haematoxylin and eosin (H&E) staining

Histopathological examination was performed to observe the microstructure of the trabecular bone and bone marrow in the GIONFH rats. After the μCT scans were completed, the femoral heads were decalcified, paraffin‐embedded and sectioned coronally at a thickness of 5 μm. The tissue sections were placed on glass microscope slides, deparaffinized and stained with haematoxylin and eosin (H&E) to evaluate the trabecular bone and bone marrow histology. Photomicrographs were acquired using a confocal microscope (Olympus).

### 2.4 Cell lines and culture conditions

Mouse RAW264.7 cells and mouse MC3T3E1 cells were obtained from the Cell Bank of the Chinese Academy of Sciences. The RAW264.7 cells were grown in high glucose medium (Gibco). The MC3T3E1 cells were grown in α‐MEM (Gibco), supplemented with penicillin‐streptomycin (Invitrogen, China) and 10% FBS (Invitrogen). All cells were grown in an incubator at 37℃ with 5% CO_2_. Transfections were performed using Lipofectamine 2000 Transfection Reagent (Invitrogen) according to the manufacturer's instructions.

### 2.5 Quantitative real‐time PCR (qRT‐PCR)

The left femoral heads were ground included all bone tissue with liquid nitrogen. Bone tissue and cell samples were harvested from each group to extract total RNA, which was performed using TRIzol reagent (Invitrogen) according to the manufacturer's instructions. The expression of non‐coding RNA and mRNA genes was examined using qRT‐PCR (SuperMix) with the housekeeping gene, β‐actin, as the internal control. Primer sequences were designed by Sangon Biotech and manufactured by AuGCT. After calibration using the β‐actin mRNA levels, the average non‐coding RNA or mRNA levels from the control or GIONFH groups were set to 1 to quantify the relative mRNA fold changes in the experimental groups.

### 2.6 Western blotting

Femoral heads and cell samples were made with the same method to qRT‐PCR. Protein was extracted using RIPA protein lysate (Solarbio) according to the manufacturer's instructions. Following sodium dodecyl sulphate (SDS) gel electrophoresis, the protein samples (40 μg) were electrotransferred to polyvinylidene difluoride (PVDF) membranes, blocked in Tris‐buffered saline with Tween (TBST) containing 5% milk and incubated at 4℃ overnight with the following primary antibodies, anti‐β‐actin (1:5,000), anti‐PRIP (1:500), anti‐NFATc1 (1:500) and anti‐BMP‐2 (1:1,000).

### 2.7 Alkaline phosphate (ALP) assay and alizarin red staining

The identification of osteoblasts was performed using alkaline phosphatase staining following the modified Kaplow's method. Mouse MC3T3E1 cells were plated in six‐well plates, with 1 × 105 cells added to each well. 1 mmoL/mL dexamethasone (Sigma) was added when the cells had adhered to the well surface. After 24 hours, the culture medium was replaced with medium containing dexamethasone (0.1 mmoL/mL), vitamin C (50 μmoL/L, Sigma) and sodium β‐glycerophosphate (10 mmol/L, Sigma) to induce differentiation into osteoblasts. The culture medium was replaced every 2 days for 12 days. The MC3T3E1 cells from one plate were digested and pyrolysed to measure the AKP/ALP activity according to the instructions of the alkaline phosphatase (AKP/ALP) activity test kit (Solarbio).

For verification purposes, the other plate of MC3T3E1 cells was cultured for 21 days, and then, alizarin red staining was carried out according to the manufacturer's instructions (Solarbio). The culture medium was discarded, and the cells were washed with sterile phosphate‐buffered saline (PBS), fixed in 95% ethanol and then washed in PBS. The fixed cells were stained with 0.1% alizarin red‐Tris‐HCI (pH 8.3) and were incubated at 37℃ for at least 1 hour before washing with double‐distilled water (ddH_2_O), dried, sealed and observed under a high‐power microscope.

### 2.8 Tartrate‐resistant acid phosphatase (TRAP) staining

The identification of osteoclasts was performed using a tartrate‐resistant acid phosphatase colorimetric assay to observe the characteristic multinucleated osteoclast morphology. Mouse RAW264.7 cells were plated in six‐well plates with each well receiving 1 × 104 cells. 1 × 10‐6 mol/L dexamethasone (Sigma) was added to each well when the cells had adhered to the well surface. After 24 hours, the culture medium was replaced with medium containing M‐CSF (30 ng/mL, Sino Biological) and RANKL (100 ng/mL, Sino Biological) to induce differentiation into osteoclasts. The culture medium was replaced every 2 days for 6 days. Then, the culture medium was discarded, and the cells were fixed using a fixative solution for 30 seconds and washed using ddH2O. Then, the cells were incubated at 37℃ under reduced light conditions for 1 hour in a mixture of ddH_2_O, Fast garnet GBC base solution, sodium nitrite solution, naphthol AS‐BI phosphate solution, acetate solution and tartrate solution. Finally, the mixture was discarded, and the cells were thoroughly washed with ddH_2_O. The nuclei were counterstained with Gill's haematoxylin for 2 minutes, washed with ammonia water and observed under a high‐power microscope.

### 2.9 Luciferase reporter assay

The mRNA sequence for PRIP that contained the putative binding sites for miR‐329‐5p was inserted into the pmirGLO dual luciferase vector (Promega) to form the reporter vector pmirGLO‐PRIP‐wild‐type (PRIP‐WT). To mutate the putative binding site for miR‐329‐5p in the PRIP gene, the putative binding site sequence was replaced by Lipofectamine 2000, as indicated, to construct pmirGLO‐PRIP‐WT or PRIP‐MUT and miR‐329‐5p mimics or miR‐NC. Meanwhile, the same protocol was performed for MALAT1 and miR‐329‐5p. The relative luciferase activity was measured using a dual luciferase reporter assay system according to the manufacturer's instructions.

### 2.10 Statistical analysis

All experiments were repeated three times independently, and the numerical data were shown as means ± standard deviations (SD) with ANOVA (Fisher's least significant difference [LSD]) method to assess significant differences between groups. The statistical analysis was performed using SPSS, version 24.0, software (IBM Corp) and GraphPad Prism, version 7.00, (GraphPad Software). A *P* value less than .05 was considered to be statistically significant.

## 3 RESULTS

### 3.1 MALAT1/miR‐329‐5p/PRIP signalling participated in the pathogenesis of GIONFH in the rat model

MRI scans, μCT and H&E staining were performed to observe the changes in the femoral head structure in the GIONFH rat model.

**FIGURE 1:**
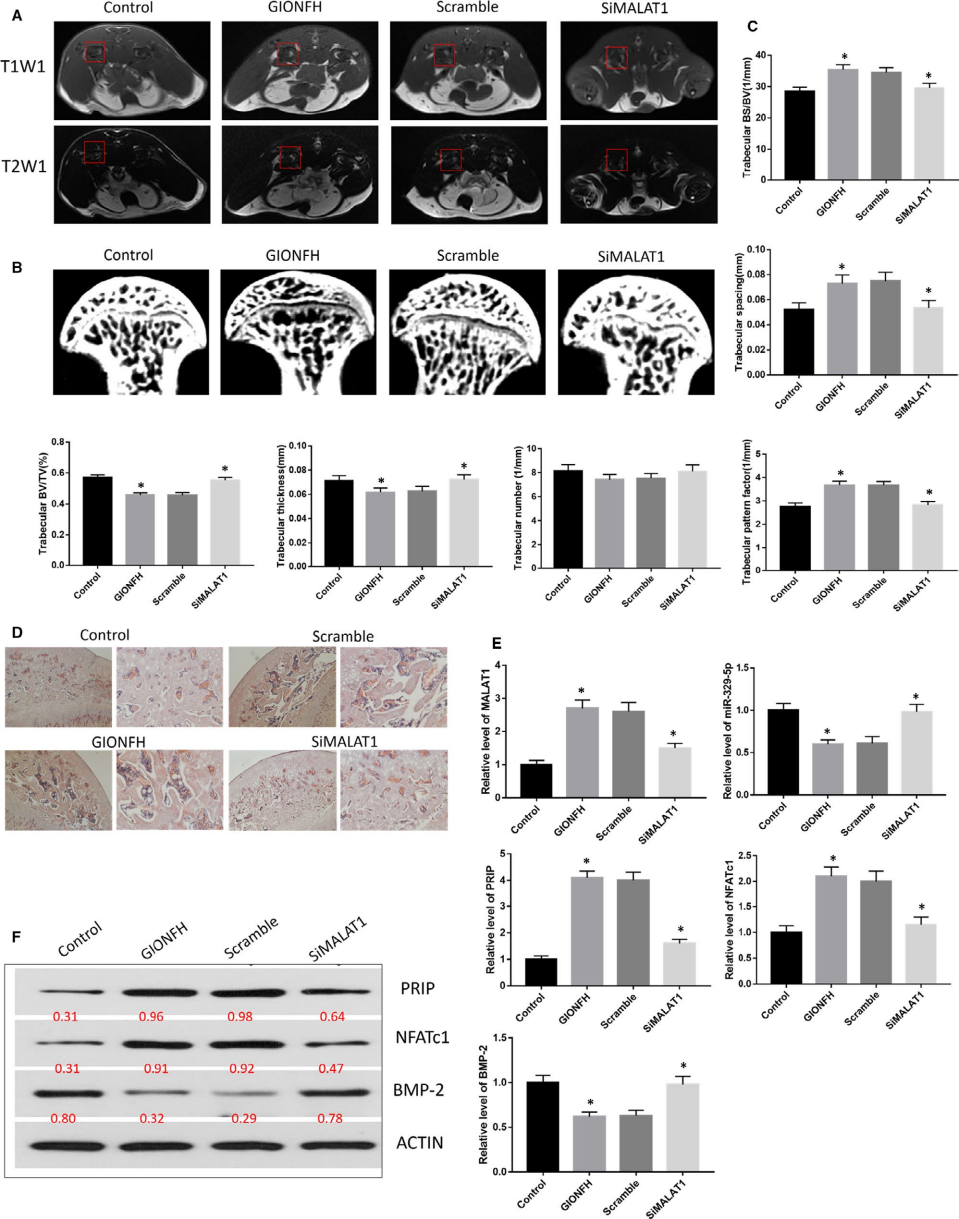
A, Representative MRI T1W1 and T2W1 scans of femoral head; the red boxed areas indicate different signal in T1W1 and T2W1 scan. B, Representative μCT scan of the femoral head. C, Quantification of trabecular bone structure within the ROI in B. Trabecular BS/BV, trabecular spacing, trabecular BV/TV, trabecular thickness, trabecular number and trabecular pattern factor. D, Representative H&E staining of trabecular at the proximal epiphysis of the femoral head. Left panel: Scale bar = 500 μm; Right panel: Scale bar = 100 μm. E, Relative expression level of LncRNA MALAT1, miR‐329‐5p, PRIP, NFATc1 and BMP‐2 in the femoral head tissues of rats. F, Western blot analysis of PRIP, NFATc1 and BMP‐2 in the femoral head tissues of rats. ^*^ *P* < .05

MRI scans revealed heterogeneous T1W1 (T1‐weighted imaging) signals and high‐intensity T2W1 (T2‐weighted imaging) signals in the femoral head in the GIONFH and scramble groups. In contrast, the siMALAT1 and control groups showed homogeneous T1W1 signals and low‐intensity T2W1 signals. These data indicated that the GIONFH rat model was successfully produced and that bone metabolism participated in the pathogenetic process of GIONFH (Figure 1A).

μCT demonstrated that the bone marrow cavities were larger at the midshaft area, and the trabeculae were thinner and fewer in the GIONFH. These changes indicated the presence of trabecular bone loss and abnormal microstructure, which was significantly improved by siMALAT1 treatment. The quantitative analysis revealed that the trabecular BS/BV, spacing and pattern factors were remarkably increased in the GIONFH group. Furthermore, these changes were significantly decreased when the GIONFH rats received siMALAT1 treatment. The trabecular BV/TV, thickness and number were remarkably decreased in the GIONFH group, and these changes were significantly improved by siMALAT1 treatment (Figure 1B,C).

Moreover, H&E staining showed that there were enlargement of the bone marrow cavity, loss and thinning of the trabeculae, empty bone lacunae and the presence of adipocytes in the bone marrow of the GIONFH group compared with the control group. All of the GIONFH effects were rescued by siMALAT1 treatment (Figure 1D).

We examined the potential mechanisms of action of the long non‐coding RNA MALAT1, microRNA 329‐5p and mRNA in glucocorticoid‐induced osteonecrosis of the femoral head. To the end, the femoral head tissues from the control, GIONFH, scramble, siMALAT1 groups were analysed using qRT‐PCR. The results showed that the levels of MALAT1, PRIP and NFATc1 were significantly up‐regulated, whereas the levels of miR‐329‐5p and BMP‐2 were significantly down‐regulated in the GIONFH and scramble groups compared to the control group. After siMALAT1 treatment, the levels of MALAT1, PRIP and NFATc1 were significantly down‐regulated, and the levels of miR‐329‐5p and BMP‐2 were significantly up‐regulated in the siMALAT1 group compared with the GIONFH group (Figure 1E).

Western blots were performed to verify the regulation of MALAT1 and PRIP, NFATc1 and BMP‐2. The results demonstrated that the expression levels of PRIP and NFATc1 were up‐regulated, whereas BMP‐2 was down‐regulated in the SIONFH group compared with the control group. When the rats were treated with siMALAT1, PRIP and NFATc1 were down‐regulated, and BMP‐2 was up‐regulated in the siMALAT1 group compared with the GIONFH and scramble groups (Figure 1F).

### 3.2 MALAT1 increased osteoclast differentiation and formation by binding to miR‐329‐5p to up‐regulate PRIP in vitro

**FIGURE 2:**
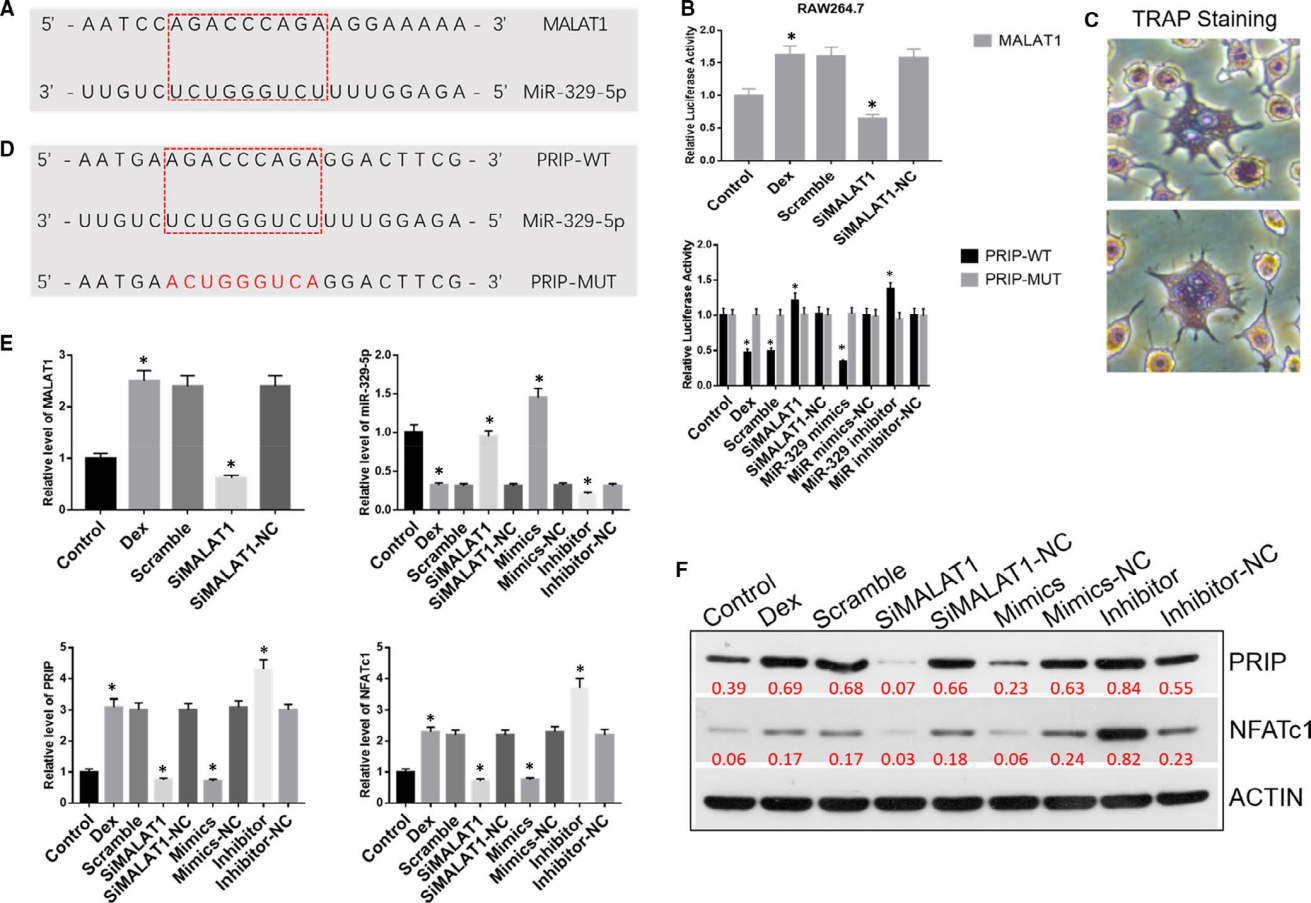
A, The binding site between LncRNA MALAT1 and miR‐329‐5p. B, Dual luciferase reporter gene assay was performed to verify the relationship between LncRNA MALAT1 and miR‐329‐5p, between PRIP and miR‐329‐5p in RAW264.7 cells. C, TRAP staining was performed to diagnosis of osteoclasts in RAW264.7 cells. D, The binding site between PRIP and miR‐329‐5p. E, Relative expression level of LncRNA MALAT1, miR‐329‐5p, PRIP and NFATc1 in RAW264.7 cells. F, Western blot analysis of PRIP and NFATc1 in RAW264.7 cells. ^*^ *P* < .05

Recently, numerous studies have shown that long non‐coding (lnc) RNAs can be used as competitive endogenous RNAs (ceRNAs) that bind to (ie sponge) miRNAs to regulate pathogenetic processes in many diseases. We predicted that miR‐329‐5p contained a putative binding site for MALAT1 using the starBase bioinformatics database (v2.0). A dual luciferase reporter gene assay demonstrated a targeting relationship between MALAT1 and miR‐329‐5p in RAW264.7 cells. We predicted that miR‐329‐5p contained a putative binding site and a higher prevalence for PRIP using TargetScan and Mouse Genome Informatics. The dual luciferase reporter gene assay revealed that miR‐329‐5p mimics decreased the luciferase activity of PRIP‐WT, but did not change the relative luciferase activity of MALAT1‐MUT in RAW264.7 cells. These results confirmed the presence of a target relationship between PRIP and miR‐329‐5p (Figure 2A,B,D).

The mouse RAW264.7 cell line was induced to differentiate into osteoclasts with exposure to M‐CSF and RANKL for six days. After TRAP staining, we observed large, multinuclear, irregular‐shaped osteoclasts under a microscope (Figure 2C).

To investigate the role of MALAT1/miR‐329‐5p/PRIP signalling in osteoclast differentiation, we used qRT‐PCR to assess RNA expression and observed that the levels of MALAT1, PRIP and NFATc1 were significantly up‐regulated, whereas the level of miR‐329‐5p was significantly down‐regulated in the dexamethasone‐treated group compared to the control group. After transfecting with siMALAT1, the expression of MALAT1, PRIP and NFATc1 was down‐regulated, and miR‐329‐5p was up‐regulated in the siMALAT1 group compared to dexamethasone and scramble groups. Next, we separately transfected miR‐329‐5p mimics and a miR‐329‐5p inhibitor into RAW264.7 cells. We observed that the level of miR‐329‐5p was remarkably increased in the mimics group and remarkably decreased in the inhibitor group compared with the dexamethasone and scramble groups. The mimics‐NC and inhibitor‐NC groups exhibited no changes compared with the dexamethasone and scramble groups. Conversely, PRIP and NFATc1 were significantly increased in the inhibitor group, decreased in the mimics group and did not change in mimics‐NC and inhibitor‐NC groups compared with the dexamethasone and scramble groups (Figure 2E).

To further verify the effect of MALAT1/miR‐329‐5p/PRIP signalling on osteoclast differentiation and formation, we detected the protein expression levels of PRIP and NFATc1 using Western blot analysis. The proteins, PRIP and NFATc1, which are proteins related to osteoclast differentiation, were up‐regulated in RAW264.7 cells exposed to dexamethasone. However, when the cells were treated with siMALAT1, the expression levels of PRIP and NFATc1 were down‐regulated compared with the dexamethasone and scramble groups. We separately transfected miR‐329‐5p mimics and miR‐329‐5p inhibitor into RAW264.7 cells. We observed that the expression levels of PRIP and NFATc1 were significantly down‐regulated in the mimics group and up‐regulated in the inhibitor group. Meanwhile, PRIP and NFATc1 did not produce any changes in the mimics‐NC and inhibitor‐NC groups, when compared with the dexamethasone and scramble groups (Figure 2F).

### 3.3 MALAT1 decreased osteoblast differentiation and formation in vitro by binding to miR‐329‐5p to up‐regulate PRIP

**FIGURE 3:**
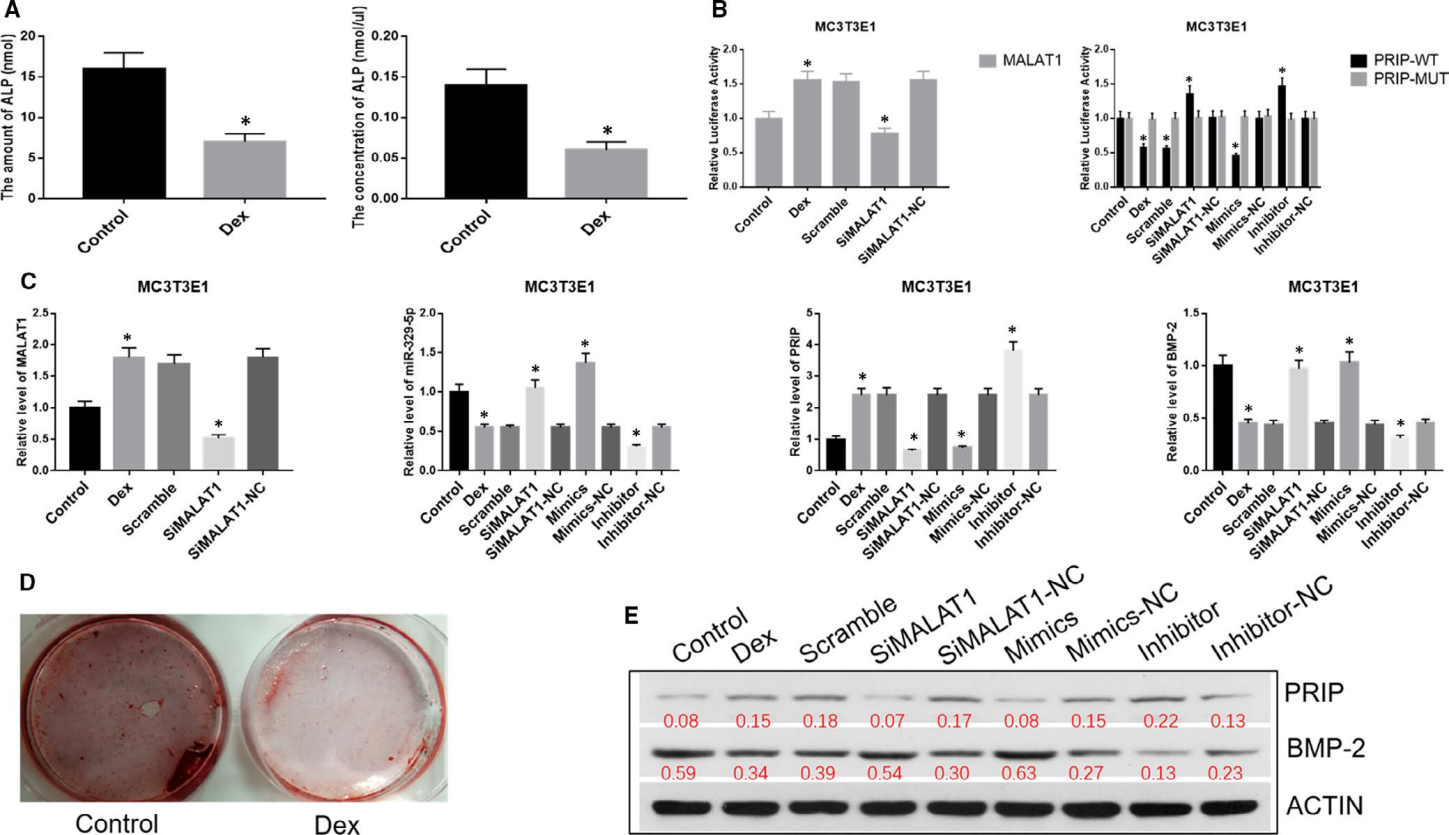
A, The amount and concentration of ALP in MC3T3E1 cells. B, Dual luciferase reporter gene assay was performed to verify the relationship between LncRNA MALAT1 and miR‐329‐5p, between PRIP and miR‐329‐5p in MC3T3E1 cells. C, Relative expression level of LncRNA MALAT1, miR‐329‐5p, PRIP and BMP‐2 in MC3T3E1 cells. D, Alizarin red staining was used to detect the osteogenesis effect of dexamethasone in MC3T3E1 cells. E, Western blot analysis of PRIP and BMP‐2 in MC3T3E1 cells. ^*^ *P* < .05

The results of the dual luciferase reporter gene assay in MC3T3E1 cells were similar to RAW264.7 cells, which demonstrated a targeting relationship for MALAT1/miR‐329‐5p and miR‐329‐5p/PRIP. The amount and concentration of ALP expression in the dexamethasone group were higher than the control group, as revealed by alizarin red staining (Figure 3A,B,D).

To investigate the role of MALAT1/miR‐329‐5p/PRIP signalling in osteoblast differentiation, we used qRT‐PCR to detect RNA expression. We found that the levels of MALAT1 and PRIP were clearly up‐regulated, and the levels of miR‐329‐5p and BMP‐2 were significantly down‐regulated in the dexamethasone group compared to the control group. After transfecting with siMALAT1, the expression of MALAT1 and PRIP was down‐regulates, and miR‐329‐5p and BMP‐2 were up‐regulated in the siMALAT1 group compared with the dexamethasone and scramble groups. We separately transfected miR‐329‐5p mimics and a miR‐329‐5p inhibitor into MC3T3E1 cells and observed that miR‐329‐5p and BMP‐2 were remarkably increased in the mimics group and decreased in the inhibitor group compared with the dexamethasone and scramble groups. The mimics‐NC and inhibitor‐NC groups did not change compared with the dexamethasone group. Conversely, the level of PRIP was significantly increased in the inhibitor group, decreased in the mimics group and did not change in the mimics‐NC and inhibitor‐NC groups compared with the dexamethasone and scramble groups (Figure 3C).

To verify the effect of MALAT1/miR‐329‐5p/PRIP signalling on osteoblast differentiation and formation, we analysed the protein expression levels of PRIP and BMP‐2 using Western blot analysis. The MC3T3E1 cells treated with dexamethasone exhibited up‐regulated PRIP and down‐regulated BMP‐2, which are proteins relating to osteoblast differentiation. However, cells treated with siMALAT1 showed down‐regulated PRIP expression levels, and BMP‐2 expression was up‐regulated compared with the dexamethasone and scramble groups. We separately transfected miR‐329‐5p mimics and a miR‐329‐5p inhibitor into MC3T3E1 cells. We observed that the expression level of PRIP was significantly down‐regulated in the mimics group and up‐regulated in the inhibitor group. However, the change in expression level for BMP‐2 was the opposite of PRIP. PRIP and NFATc1 did not exhibit any change in the mimics‐NC and inhibitor‐NC groups when compared with the dexamethasone and scramble groups (Figure 3E).

### 3.4 Overweight‐loading conditions aggravated the pathological progress of GIONFH by MALAT1/miR‐329‐5p/PRIP signalling in vivo

**FIGURE 4:**
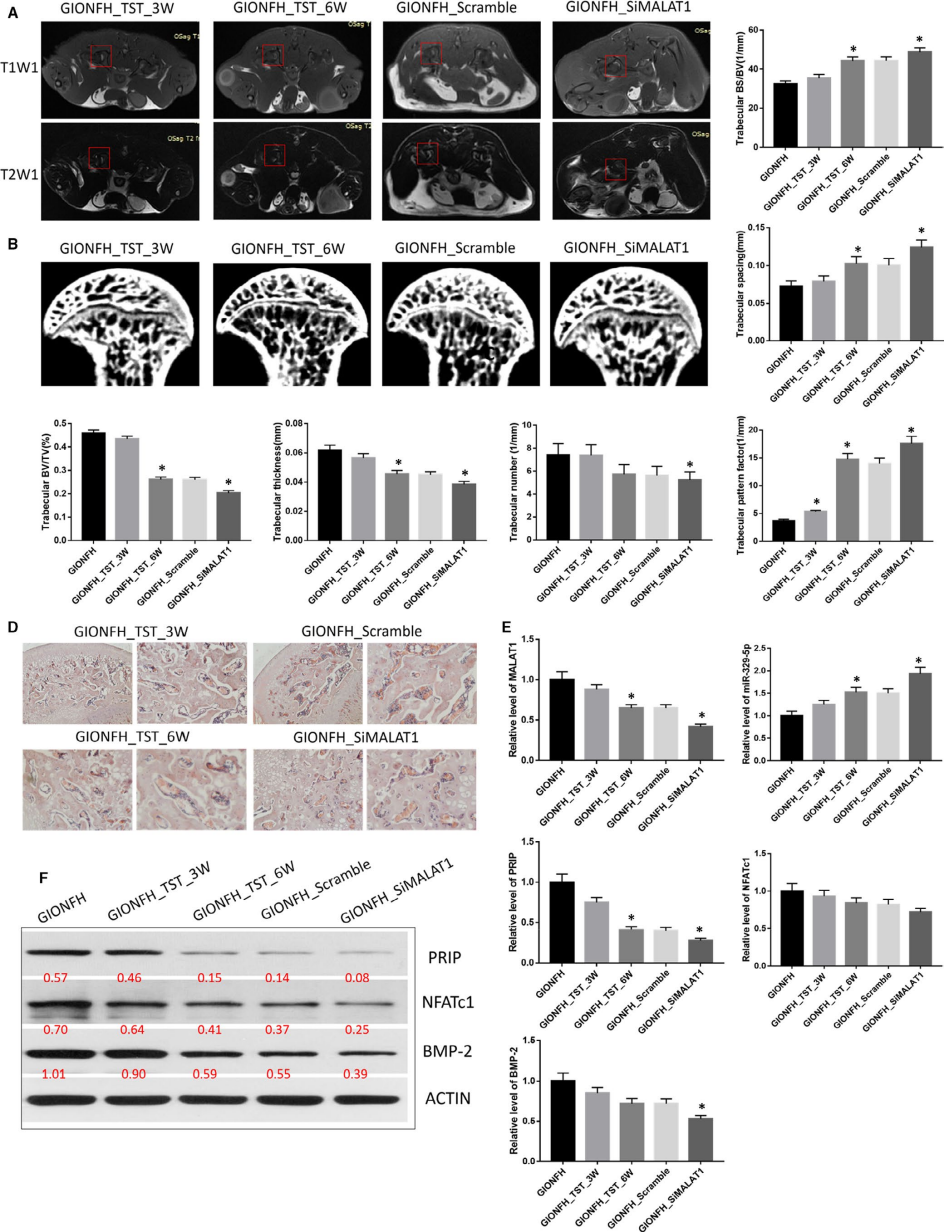
A, Representative MRI T1W1 and T2W1 scans of femoral head in tail suspension test group; the red boxed areas indicate different signal in T1W1 and T2W1 scan. B, Representative μCT scan of the femoral head in tail suspension test group. C, Quantification of trabecular bone structure within the ROI in B. Trabecular BS/BV, trabecular spacing, trabecular BV/TV, trabecular thickness, trabecular number and trabecular pattern factor. D, Representative H&E staining of trabecular at the proximal epiphysis of the femoral head in tail suspension test group. Left panel: Scale bar = 500 μm; Right panel: Scale bar = 100 μm. E, Relative expression level of LncRNA MALAT1, miR‐329‐5p, PRIP, NFATc1 and BMP‐2 in the femoral head tissues in tail suspension test group. F, Western blot analysis of PRIP, NFATc1 and BMP‐2 in the femoral head tissues in tail suspension test group. ^*^ *P* < .05

MRI scans revealed that the heterogeneous T1W1 signals and high‐intensity T2W1 signals in the femoral head were aggravated in both GIONFH_Treadmill_2W and GIONFH_Treadmill_4W rats. The observed changes were more severe in the fourth week. In contrast, the GIONFH_siMALAT1 group exhibited a weaker homogeneous T1W1 signal and a low‐intensity T2W1 signal. μCT demonstrated that the bone marrow cavities and trabeculae presented abnormal microstructure. Specifically, the bone formation was abnormal in the loading area, and the empty spaces were larger in the unloaded area, for both GIONFH_Treadmill_2W and GIONFH_Treadmill_4W rats, and the changes were more severe in the fourth week. These changes were dramatically improved with siMALAT1 treatment. The quantitative analysis revealed that the trabecular BS/BV, spacing, pattern factors were remarkably increased in the GIONFH_Treadmill group. However, these changes were significantly decreased when rats were given siMALAT1 treatment. We also observed that the trabecular BV/TV, thickness and number were notably decreased in the GIONFH_Treadmill group, and these changes were significantly improved with siMALAT1 treatment. Concurrently, similar changes in the microstructure of the femoral head were revealed using H&E staining (Figure 4A,B,C,D).

To evaluate the potential mechanisms of action of MALAT1, miR‐329‐5p and mRNA in GIONFH, femoral head tissues from all groups were analysed using qRT‐PCR. The results demonstrated that the levels of MALAT1, PRIP, NFATc1 and BMP‐2 were significantly up‐regulated, and the level of miR‐329‐5p was significantly down‐regulated in the GIONFH_Treadmill_4W and scramble groups compared to that of the GIONFH group. When treated with siMALAT1, the levels of MALAT1, PRIP, NFATc1 and BMP‐2 were significantly down‐regulated, and the level of miR‐329‐5p was significantly up‐regulated in the GIONFH_siMALAT1 group compared with the GIONFH_Treadmill_4W group. Also, Western blotting was used to verify the regulation by MALAT1 for PRIP, NFATc1 and BMP‐2. The results showed that the expression levels of PRIP, NFATc1 and BMP‐2 were up‐regulated in the GIONFH_Treadmill and scramble groups, and were reduced by siMALAT1 treatment (Figure 4E,F).

### 3.5 Unloading conditions delayed the pathological progress of GIONFH by MALAT1/miR‐329‐5p/PRIP signalling in vivo

**FIGURE 5:**
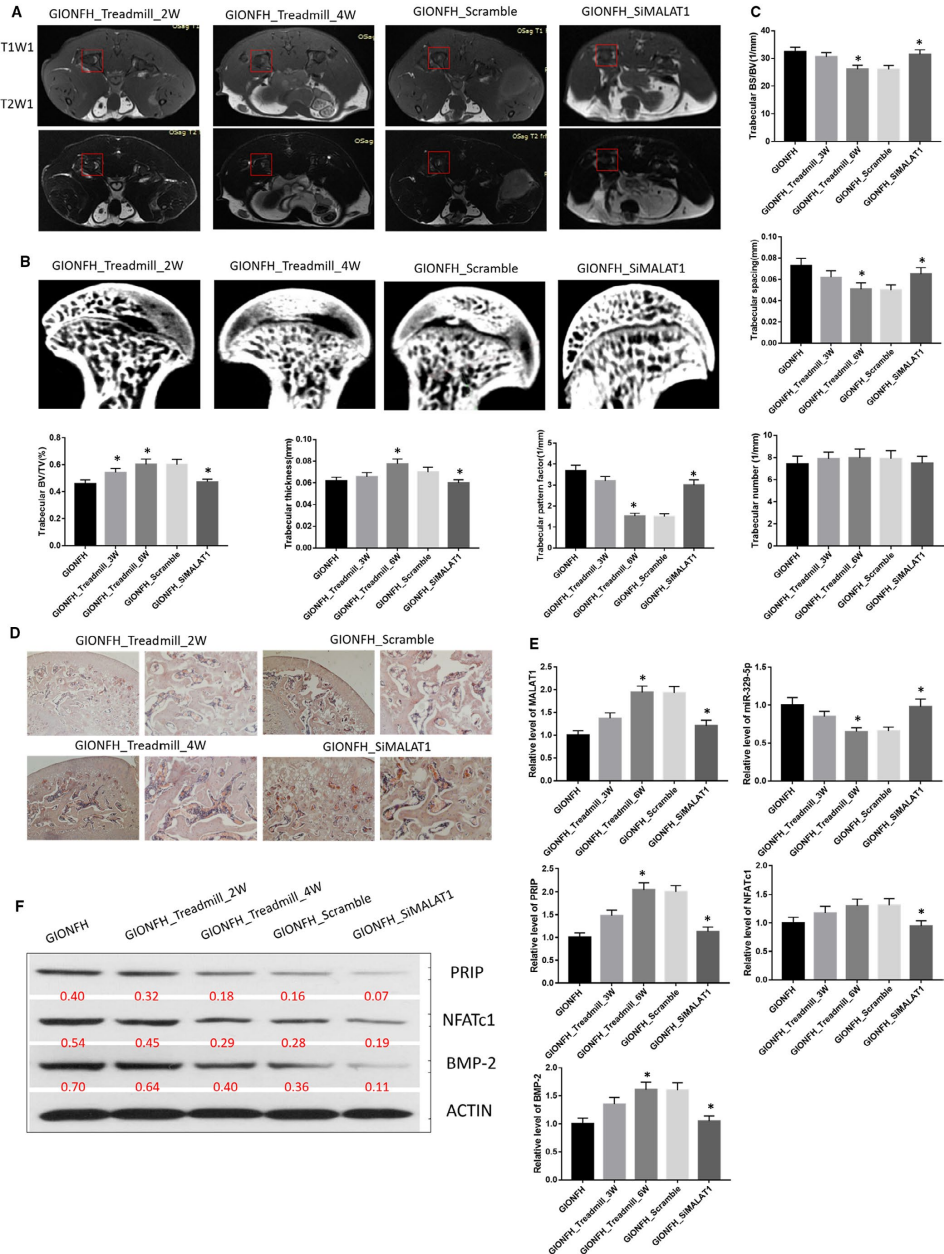
A, Representative MRI T1W1 and T2W1 scans of femoral head in Treadmill group; the red boxed areas indicate different signal in T1W1 and T2W1 scan. B, Representative μCT scan of the femoral head in treadmill group. C, Quantification of trabecular bone structure within the ROI in B. trabecular BS/BV, trabecular spacing, trabecular BV/TV, trabecular thickness, trabecular number and trabecular pattern factor. D, Representative H&E staining of trabecular at the proximal epiphysis of the femoral head in Treadmill group. Left panel: Scale bar = 500 μm; Right panel: Scale bar = 100 μm. E, Relative expression level of LncRNA MALAT1, miR‐329‐5p, PRIP, NFATc1 and BMP‐2 in the femoral head tissues in Treadmill group. F, Western blot analysis of PRIP, NFATc1 and BMP‐2 in the femoral head tissues in Treadmill group. ^*^ *P* < .05

MRI scans showed heterogeneous T1W1 signals and high‐intensity T2W1 signals in the femoral head in the GIONFH and scramble groups. In contrast, the siMALAT1 and TST groups exhibited a homogeneous T1W1 signal and a low‐intensity T2W1 signal. μCT demonstrated that the distribution of the bone marrow cavity and trabeculae were more uniform, the trabeculae were thinner in the GIONFH_TST and GIONFH_siMALAT1 groups compared with the GIONFH group. The quantitative analysis revealed that the trabecular BS/BV, spacing and pattern factors were remarkably increased, and the trabecular BV/TV, thickness and number were decreased in the GIONFH_TST_6W and GIONFH_siMALAT1 groups compared with the GIONFH group. The same results also were observed in the H&E stained tissue sections (Figure 5A‐D).

To evaluate the potential mechanism of MALAT1, miR‐329‐5p and mRNA in GIONFH, the femoral head tissues from different groups were analysed using qRT‐PCR. The results showed that the levels of MALAT1 and PRIP were significantly down‐regulated, and the level of miR‐329‐5p was significantly up‐regulated in the GIONFH_TST and GIONFH_siMALAT1 groups compared with the GIONFH group. The levels of NFATc1 and BMP‐2 exhibited no changes among the different groups. The changes of PRIP, NFATc1 and BMP‐2 were verified using Western blotting (Figure 5E,F).

## 4 DISCUSSION

Our results revealed a direct effect of biomechanical forces on the pathogenesis of GIONFH through MALAT1/miR‐329‐5p/PRIP signalling. This conclusion was based on the following information. First, we observed that the bone mineral density was significantly decreased in the GIONFH rat model using μCT imaging and H&E staining. These results indicated that abnormal bone metabolism played a critical role in the pathogenesis of GIONFH. Second, the expression of MALAT1, miR‐329‐5p, PRIP, NFATc1 and BMP‐2, which are all related to bone metabolism, were significantly different in the GIONFH group compared to the control group, and these effects could be reversed by siMALAT1 treatment. Third, we confirmed the presence of a targeting relationship for MALAT1/miR‐329‐5p and miR‐329‐5p/PRIP through a bioinformatics database and a dual luciferase reporter gene assay. Fourth, MALAT1 promoted osteoclast differentiation and inhibited osteoblast differentiation by binding to or ‘sponging’ miR‐329‐5p to up‐regulate PRIP in vitro. Fifth, overweight‐loading conditions aggravated the pathological progress of GIONFH, whereas unloading conditions delayed the pathogenesis of GIONFH through MALAT1/miR‐329‐5p/PRIP signalling in vivo.

Long non‐coding (Lnc) RNAs, containing more 200 nucleotides, play important roles in the pathogenesis of diseases through regulating gene expression. In 2019, Xiang et al^12^ found that lncRNA RP11‐154D6 might contribute to the progression of steroid‐induced ONFH though regulating the behaviour of BMSCs in vivo. This observation might provide new insights into the pathogenesis and treatment for GIONFH. In 2018, Wang et al^11^ showed that specific lncRNA expression profiles were closely associated with abnormal adipogenic and osteogenic transdifferentiation of BMSCs during the pathogenesis of GIONFH.

Metastasis‐associated lung adenocarcinoma transcript 1 (MALAT1) is a lncRNA reported to promote osteosarcoma development through regulation of HMGB1 via miR‐142a‐3p and miR‐129a‐5p.^13^ Also, miR‐329 suppresses osteosarcoma development by down‐regulating Rab10.^14^ However, MALAT1 and miR‐329‐5p had never been reported in previous studies of GIONFH. We predicted that miR‐329‐5p contained a putative binding site for MALAT1 based on information from a bioinformatics database, and qRT‐PCR demonstrated a significant difference in the level of MALAT1 and miR‐329‐5p in vitro and in vivo. We further confirmed the presence of a targeting relationship between MALAT1 and miR‐329‐5p using a dual luciferase reporter gene assay in RAW264.7 cells and MC3T3E1 cells, which indicated that LncRNA MALAT1 may bind to miR‐329‐5p by a putative binding site to down‐regulate the function of miR‐329‐5p. To sum up, MALAT1 might play a role in the pathogenesis of glucocorticoid‐induced osteonecrosis of the femoral head by sponging miR‐329‐5p.

In previous researches, tail suspension test was widely used to mimic the effect of microgravity and treadmill running exercise was used to mimic the overweight exercise, which were significant to research the biomechanical forces in animal experiment.^20^, ^21^ In our research, we separately used tail suspension test or treadmill running exercise to realize the unloading model or the hyper‐weight model for researching the mechanism of biomechanical forces on the osteonecrosis of the femoral head.

We found that BMD levels varied among the different experimental groups, which indicated that both osteoclasts and osteoblasts participated in the pathogenesis of GIONFH. Similarly, numerous studies also have reported on the effects of osteoclasts and osteoblasts. Phospholipase C‐related but catalytically inactive protein (PRIP) is a novel inositol 1,4,5‐trisphosphate‐binding protein with a domain organization similar to phospholipase C‐δ but lacks phospholipase activity. Harada et al^15^ found that PRIP played an important role in regulating ins (1,4,5) P3‐mediated Ca^2+^ signalling by modulating type 1 inositol polyphosphate 5‐phosphatase activity. They used PRIP knockout (KO) mice to discover that the modulation occurred through binding to ins (1,4,5) P3.

In 2015, Japanese researchers^17^ demonstrated that PRIP was implicated in BMP‐induced osteoblast differentiation through negative regulation of Smad phosphorylation, which resulted from methylation of inhibitory Smad6. In 2017, these researchers demonstrated that PRIP deficiency impaired osteoclast differentiation, particularly at the early stages, and that PRIP stimulated osteoclast differentiation though calcium‐calcineurin‐NFATc1 signalling via regulation of intracellular Ca2+ in PRIP‐KO osteoclasts.^18^ We found that lncRNA MALAT1 and miR‐329‐5p were present in osteosarcoma and regulated proliferation and metabolism of osteosarcoma cells.^13^, ^14^ Abnormal bone metabolism is one theory for the pathogenesis of GIONFH. Previous studies have usually focused on either osteoclasts or osteoblasts since these cells are regulated through different signalling pathways.^10^, ^22^, ^23^, ^24^ Our study is the first to assess the effect of concurrent osteoclast and osteoblast differentiation on GIONFH through the expression of PRIP protein. In our study, the expression of PRIP was up‐regulated in the GIONFH and GIONFH_Treadmill groups in vivo, and when RAW264.7 cells or MC3T3E1 cells were treated with dexamethasone in vitro.

There still is no effective treatment for glucocorticoid‐induced osteonecrosis of the femoral head. It is common to use unloading conditions in the early phase of GIONFH, which can delay or even reduce the pathological processes that occur in GIONFH. However, although many orthopaedic doctors have recognized that biomechanical forces might participate in the pathogenesis of GIONFH, research specifically focusing on biomechanics and GIONFH has been meagre. Some research has revealed that the areas of osteonecrosis of the femoral head are distributed in the stress concentration zone and not the zone with increased blood supply.^25^, ^26^ The induction of a sclerosis rim was determined to be the result of bone repair and could result in effective mechanical support.^27^, ^28^ As we know, running or sports can increase bone mineral density and bone strength. On the other hand, the bone mineral density and bone strength are decreased with a lack of physical activity.^29^, ^30^ Therefore, we concluded that biomechanical forces have a vital effect on GIONFH by influencing bone metabolism.

In our results, we determined that the bone mineral density in the GIONFH group was lower than the control group. We used the tail suspension test to simulate unloading conditions in this study and observed that the empty bone space was significantly less in the TST_6W group, which indicated the pathogenesis of GIONFH was blocked. Also, the BMD was lower because low or no physical activity could decrease osteoblast activity and increase osteoclast activity. qRT‐PCR and Western blot analysis showed that BMP‐2 and NFATc1 did not exhibit different expression levels when compared with the GIONFH group. On the other hand, when GIONFH rats were allowed to exercise on a treadmill to simulate the overweight‐loading condition, we observed that the bone marrow cavities and trabeculae formed abnormal microstructures. Specifically, bone formation was increased but in the wrong loading area. Also, the empty bone space was larger in the unloading area in the GIONFH_Treadmill group, and the results were more severe in the fourth week. These changes were significantly improved by siMALAT1 treatment.

We speculated that it was difficult to observe microfractures or collapse of the femoral head due to the differences between rats and humans. Rats have a more robust ability to repair bone injury compared to humans. Consequently, we thought that the abnormal microstructure of the femoral head in the model rats was equivalent to microfractures or collapse of the femoral head in humans with GIONFH.

Thus, we concluded that the overweight‐loading condition aggravated the pathogenesis of GIONFH. Concurrently, the abnormal changes in the femoral head were reduced by siMALAT1 treatment. Finally, we concluded that the overweight‐loading condition aggravated the pathogenesis of GIONFH, and the unloading condition blocked the pathogenesis of GIONFH through MALAT1/miR‐329‐5p signalling.

Currently, glucocorticoid‐induced osteonecrosis of the femoral head is an urgent orthopaedic problem worldwide. However, the pathogenesis of GIONFH is unclear. Recently, several investigators proposed a ‘multi‐hit hypothesis’,^1^, ^31^, ^32^ in which no single mechanism was sufficient to result in GIONFH. They proposed that there several factors or triggers were necessary to sufficiently deprive the bone of nutrients to cause osteonecrosis. In our investigation, we combined abnormal bone metabolism, biomechanical forces and non‐coding RNAs to study the pathogenesis of GIONFH. We also considered the role of osteoclasts and osteoblasts together in the abnormal bone metabolism associated with the pathogenesis of GIONFH. We confirmed that these factors were involved in the pathogenesis of GIONFH. In the future, we will combine osteoimmunology, assessment of apoptosis, lipid metabolism, endothelial cell damage, coagulation and oxidative stress to further elucidate the pathogenesis of GIONFH. We also will search for new therapeutic methods that can be used to cure patients with GIONFH.

**FIGURE 6:**
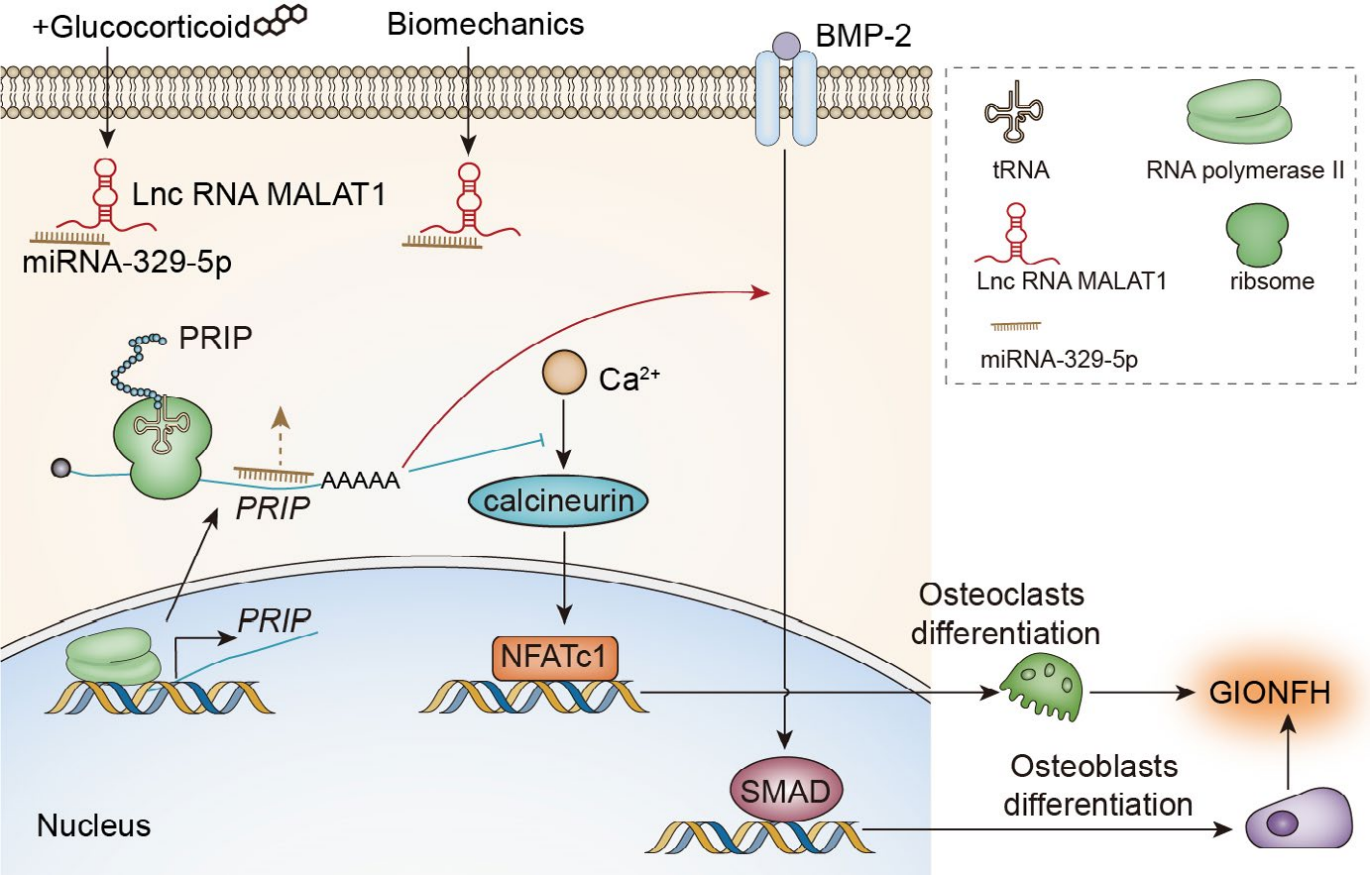
Schematic illustration of the effect of the biomechanics and MALAT1/miR‐329‐5p/PRIP signalling on GIONFH pathogenesis

In conclusion, MALAT1 played a vital role in the pathogenesis of glucocorticoid‐induced osteonecrosis of the femoral head by sponging miR‐329‐5p to up‐regulate PRIP. We also determined that biomechanical forces could aggravate the pathogenesis of GIONFH through MALAT1/miR‐329‐5p/PRIP signalling (Figure 6).

## CONFLICT OF INTEREST

The authors confirm that there are no conflicts of interest.

## AUTHOR CONTRIBUTIONS

**Guomin Li:** Data curation (equal); Investigation (equal); Writing‐review & editing (equal). **Bing Li:** Formal analysis (equal); Methodology (equal). **Bo Li:** Resources (equal); Software (equal). **Jie Zhao:** Conceptualization (equal); Project administration (equal). **Xiaoquan Wang:** Writing‐original draft (equal). **Rui Luo:** Visualization (equal). **Yankun Li:** Supervision (equal); Validation (equal). **Jun Liu:** Funding acquisition (equal). **Ruyin Hu:** Conceptualization (equal); Resources (equal).
